# Virtual reality hip arthroscopy simulator demonstrates sufficient face validity

**DOI:** 10.1007/s00167-018-5038-8

**Published:** 2018-07-11

**Authors:** Jonathan D. Bartlett, John E. Lawrence, Vikas Khanduja

**Affiliations:** 1 School of Clinical Medicine, University of Cambridge, Hills Road, Cambridge, CB2 0QQ UK; 2 Department of Trauma and Orthopaedics, Addenbrooke’s - Cambridge University Hospital, Hills Road, Cambridge, CB2 0QQ UK

**Keywords:** Virtual reality, Simulation, Hip arthroscopy, Face validity

## Abstract

**Purpose:**

To test the face validity of the hip diagnostics module of a virtual reality hip arthroscopy simulator.

**Methods:**

A total of 25 orthopaedic surgeons, 7 faculty members and 18 orthopaedic residents, performed diagnostic supine hip arthroscopies of a healthy virtual reality hip joint using a 70° arthroscope. Twelve specific targets were visualised within the central compartment; six via the anterior portal, three via the anterolateral portal and three via the posterolateral portal. This task was immediately followed by a questionnaire regarding the realism and training capability of the system. This consisted of seven questions addressing the verisimilitude of the simulator and five questions addressing the training environment of the simulator. Each question consisted of a statement stem and 10-point Likert scale. Following similar work in surgical simulators, a rating of 7 or above was considered an acceptable level of realism.

**Results:**

The diagnostic hip arthroscopy module was found to have an acceptable level of realism in all domains apart from the tactile feedback received from the soft tissue. 23 out of 25 participants (92%) felt the simulator provided a non-threatening learning environment and 22 participants (88%) stated they enjoyed using the simulator. It was most frequently agreed that the level of trainees who would benefit most from the simulator were registrars and fellows (22 participants; 88%). Additionally, 21 of the participants (84%) agreed that this would be a beneficial training modality for foundation and core trainees, and 20 participants (80%) agreed that his would be beneficial for consultants.

**Conclusions:**

This VR hip arthroscopy simulator was demonstrated to have a sufficient level of realism, thus establishing its face validity. These results suggest this simulator has sufficient realism for use in the acquisition of basic arthroscopic skills and supports its use in orthopaedics surgical training.

**Level of evidence:**

I.

**Electronic supplementary material:**

The online version of this article (10.1007/s00167-018-5038-8) contains supplementary material, which is available to authorized users.

## Introduction

Over the last decade, there has been increasing investigation into the use of virtual reality (VR) simulation in surgical education [1]. The technically demanding nature of hip arthroscopy, combined with reductions in trainee operating time, has led to steep learning curves in modern orthopaedic surgery [2, 6, 7, 14, 15, 18, 20]. Additionally, multiple studies have highlighted worse outcomes and increased operating time in arthroscopic operations performed by inexperienced surgeons, demonstrating higher incidences of chondral damage and perineal injuries in hip arthroscopies performed in the first 3 years of an arthroscopic surgeon’s training [9, 18, 21]. These increased complication rates and longer operating times mandate a strategy to overcome this initial learning curve.

VR describes the computer-generated simulation of three-dimensional environments that can be interacted with in a seemingly real or physical way. Advances in this field have led to a rapid expansion in the number of commercially available surgical simulators, with more than 400 models currently available [29]. The proposed benefit of VR simulation is to enable surgeons to become ‘pre-trained’, thus ensuring they are functioning below their attentional capacity threshold (the limit to the amount of information individuals can comprehend and address at any given time) when operating, resulting in improved safety [11].

However, before introducing VR simulators into orthopaedic surgical training systems, it is important that they are thoroughly validated. One such type of validity is face validity. This describes the verisimilitude and appropriateness of the simulator’s psychomotor fidelity and is assessed through responses to questionnaires by surgeon’s [1]. Although this is a subjective measure of a simulators’ ability to realistically recreate a procedure, it is an important step that must be taken to justify the financial investment of VR simulators.

The purpose of this study was to assess the face validity of a hip arthroscopy simulator and determine surgeons’ opinions regarding who might benefit from training with this simulator. It was hypothesised that this simulator would demonstrate a sufficient level of realism, thus establishing face validity, and that participants would find it an enjoyable and non-threatening learning environment. This is the first study to assess the face validity of a VR hip arthroscopy simulator, and to assess trainee’s attitudes to the use of VR in the acquisition of basic hip arthroscopy skills.

## Materials and methods

### Simulator

For this study, the Simbionix Arthro Mentor (3D Systems, Littleton, USA) VR simulator was used. This simulator consists of a computer with monitor, a mannequin, and two haptic feedback devices that provide tactile feedback to a pair of instruments via connecting motors. The mannequin has four predefined 5-mm arthroscopy portals at the modified anterior, anterior, anterolateral, and posterolateral sites. The image of the virtual joint was displayed on the monitor in response to the camera movements of the operator.

### Participants

For this study, 25 orthopaedic surgeons were recruited voluntarily after 2 days of a training course in arthroscopic hip surgery for orthopaedic residents in January 2017. The course was aimed at trainees with minimal or no experience in hip arthroscopy. The study was open to all surgeons participating in the course, all of whom had an interest in arthroscopy and sport medicine. No surgeons declined to participate in the study. This included 18 surgical residents (surgeons undertaking a 6-year training program in orthopaedic surgery) from variety of training programmes across Europe, as well as seven faculty members from the course (orthopaedic surgeons who have successfully completed a training programme and were experienced  hip arthroscopists). No participants had previous experience using virtual reality surgical simulators of any kind. The course spanned 2 days and covered topics including the equipment used in hip arthroscopy, the placement of arthroscopy portals (for both the lateral and supine positions), the anatomy of the hip joint and its implications in hip arthroscopy, indications for hip arthroscopy, and arthroscopic technique.

### Protocol

Each participant performed a basic diagnostic hip arthroscopy task. The task involved locating a series of 12 consecutive targets within the hip joint using an arthroscope. Six targets within the central compartment were visualised via the anterolateral portal, three via the anterior, and three via the posterolateral portal (Table 1). No targets were visualised in the peripheral compartment, as the simulator is only capable of simulating the central compartment. The task began upon insertion of the arthroscope into any of the three portals, at which point the anatomical location of the first target was displayed to the participant on-screen. Participants were required to place each target in the centre of the monitor for 3 s before the location of the next target in the examination sequence was displayed to them (ESM Video 1). Target order was identical for each participant. Before participation, all participants received an identical standardised introduction by the same individual (JB). In this, participants were introduced to the VR simulator, were explained the various modules available, and shown a demonstration of the full diagnostic examination of the hip joint on the simulator. To avoid conflict of interest, the simulation sessions were conducted in a side room with only the simulator and no company representatives present.

**Table 1:** Targets visualised during simulation task

Portal site	Target to be visualised during task
Anterolateral	Posterior transverse ligament Posterior labrum Anterior triangle Anterior labrum Posterior capsule Femoral head
Anterior	Ligamentum teres Posterior transverse ligament Anterior transverse ligament
Posterolateral	Weight-bearing acetabulum Posterior superior labrum Femoral head

### Questionnaire

Upon completion of the task, all participants were asked to complete an anonymous questionnaire regarding the realism and training capability of the system. The questionnaire consisted of seven questions addressing the verisimilitude of the simulator and five questions addressing the training environment of the simulator. This questionnaire was derived from a questionnaire previously used to assess the face validity of an arthroscopic simulator and was approved by three senior consultant arthroscopic hip surgeons to ensure its appropriateness [13]. Each question consisted of a statement stem and a Likert scale—a technique widely used in validity studies [32]. The Likert scale consisted of 10 points between “1—strongly disagree” and “10—strongly agree” in all questions and participants were asked to indicate their opinion regarding the statement stem. Furthermore, participants were given the option to provide feedback and comments at end of the questionnaire. Though there is currently no validated scoring system for assessing face validity, following similar work in surgical simulators, a rating of 7 or above was considered an acceptable level of realism [32].

### Statistical analysis

Likert scale responses were treated as ordinal and reported as percentages of responses in agreement with statements. Statistical analysis was performed with version 3.2 of R (Foundation for Statistical Computing, Vienna, Austria). As face validity is a qualitative measure, a power calculation could not be performed. The sample size used in this study is in-keeping with previous studies of face validity [10, 13, 25, 30–32].

## Results

### Realism

This simulator was found to have an acceptably high degree of realism in all parameters apart from the tactile feedback received from the soft tissue (Table 2). 20 (80%) of the surgeons who participated agreed (Likert score of ≥ 7) that the external instrumentation of simulator was realistic. 21 (84%) agreed that the visual representation of the hip joint was realistic and 20 (80%) agreed the visual representation of the instruments on the screen was realistic. 16 of the responders (64%) believed the tactile feedback from the bone to be realistic, whilst only 12 (48%) believed the tactile feedback from the soft tissue to be realistic. 18 (72%) agreed that the arthroscopy procedure was realistic, that the steps performed accurately reflected the steps taken during the actual procedure, and that the simulator gave a sense of what arthroscopy was like.

**Table 2:** Summary of the face validity questionnaire responses

Statement stem	% agreement (Likert score of ≥ 7)
Realism	
The external instrumentation was realistic	80
The visual experience of arthroscopy was realistic	84
The visual experience of the instruments on the screen was realistic	80
The feel of the bone was realistic	64
The feel of the soft tissue was realistic	48
The arthroscopy procedure was realistic	72
The steps performed in the simulator accurately reflected the steps taken during the actual procedure	72
The simulator gave a sense of what arthroscopy would be like	76
Training environment	
The simulator provided a non-threatening learning environment	92
I enjoyed using the simulator	88
The simulator is a useful training tool for foundation and core trainees (equivalent to intern level)	84
The simulator is a useful training tool for registrars and fellows (equivalent to resident level)	88
The simulator is a useful training tool for consultants (equivalent to attending level)	80

### Training experience

23 of the participants (92%) agreed that the simulator provided a non-threatening learning environment and 22 (88%) stated that they enjoyed using the simulator. It was most frequently agreed that level of trainees who would benefit from training with this simulator (not limited to the visualisation module) were registrars and fellows (equivalent to resident level) (22 participants; 88%). However, 21 (84%) of the participants agreed that this simulator would be a beneficial training modality for foundation and core trainees (equivalent to intern level), and 20 (80%) agreed that training on this simulator would be beneficial for consultants (equivalent to attending level).

## Discussion

The most important finding of this study was that the VR arthroscopic simulator tested demonstrated an adequate degree of subjective realism, thus establishing face validity. These results mimic those of similar studies relating to the use of VR simulation in orthopaedic training and support this hip arthroscopy simulator’s use in helping trainees gain basic experience in hip arthroscopy [3, 8, 10, 13, 25, 30, 31]. Previous work has demonstrated construct validity for this simulator, shown by its ability to distinguish between ‘experts’ and ‘novices’ [16, 17, 22, 24, 25, 31]. These results, taken together, support an argument for the implementation of VR simulators like this in surgical training.

Face validity has previously been demonstrated for several VR simulators of knee and shoulder arthroscopy. Work by Stunt et al. demonstrated face validity for the PASSPORT V2 training environment (Medishield B.V., Delft, the Netherlands), a knee arthroscopy VR simulator, with highly positive responses regarding realism, educational value, and user-friendliness [31]. Similarly, face validity for the Virtamed ArthroS™ simulator (VirtaMed AG, Zurich, Switzerland) has been demonstrated in two studies assessing this simulator’s knee and shoulder arthroscopy VR environments [13, 30].

From this study, tactile feedback from intra-articular structures appears to be a limiting factor in the realism of the simulated hip joint. These results complement those of previous studies that have shown other simulators to achieve mid-scale Likert scores for face validity regarding the bone and soft tissue tactile feedback [10, 13].

The primary limitation of this study is the subjective nature of face validity. The verisimilitude of the aesthetics and haptics of the simulator cannot be objectively measured and are therefore open to interpretation. Positive or negative biases regarding simulator use may have influenced the participants’ responses and cannot be controlled for. However, these results do highlight the potential benefit of VR simulator training in orthopaedic surgery.

Though the questionnaire utilised in this study was derived from one previously used to assess the face validity of arthroscopic simulators, no such questionnaires have been formally validated in the assessment of face validity. Additionally, due to limited resources it was not possible to test this questionnaire on a pilot population before its use. Instead the expert opinion of three consultant hip arthroscopic surgeons was utilised to assess its appropriateness.

Furthermore, the influence of a recruitment bias based on the individuals who participated in this study cannot be excluded—18 of the 25 participants were surgical residents undergoing orthopaedic training, with an interest in hip arthroscopy. As such, these individuals may have over-valued the utility of these simulators in training due to reductions in operating time in surgical training and personal interests in the simulated procedures. These participants also had less arthroscopic experience compared to the seven faculty members—all of whom were expert hip arthroscopists. This may have hindered their ability to accurately assess the subjective realism of the simulator.

Additionally, due to time constraints, participants were only able to perform a single diagnostic hip arthroscopy task on the simulator. Other training modules, including probe examination and pathological identification, were not assessed and therefore any conclusions drawn regarding the face validity of the simulator are restricted to the diagnostic hip arthroscopy task. This is of particular importance with regards to the tactile feedback from the simulated structures. Though this study found that only 64 and 48% of participants felt the bone and soft tissue structures provided realistic haptic feedback, respectively, we were unable to investigate the realism of this feedback in the simulated probing modules. Further investigation of the face validity of these more complex tasks is necessary before widespread conclusions can be drawn. Furthermore, the knee and shoulder arthroscopy simulation modalities of this simulator have not been analysed and therefore have not been validated.

Another limitation of this study is that it is unable to demonstrate any measurable benefits to the trainee. If hospitals and training centres are to justify the investment of VR simulators, measurable and cost-efficient benefits to trainee’s operating room performance must be established. Such studies have been performed for several other simulators, demonstrating objective improvements in operating room performance by individuals trained on knee and shoulder simulators, when compared to untrained controls [4, 5, 26, 33]. Unfortunately, no such studies have been conducted with regards to hip arthroscopy and this should be an area of future investigation. Additionally, more robust analysis of these benefits has been conducted for a number of surgical simulators in different specialities leading to the implementation of these simulators in surgical training [12, 23, 27, 28]. This analysis has also extended to the cost benefits of simulator implementation, including cost recovered from reduced procedure time and reduced complication rates [19]. Evidence of such benefits is currently lacking with orthopaedic simulators and it is therefore unclear if orthopaedic surgical simulators are a cost-effective investment.

The demonstration of face validity for this virtual reality hip arthroscopy simulator supports its use in the acquisition of basic arthroscopic skills in orthopaedic surgical training. Such use has been theorised to reduce learning curves of procedures, improve patient safety and increase operative efficiency [11, 19]. As such, demonstration of sufficient realism is an important step that must be taken to justify the financial investment of VR simulators. However, demonstration of real-world improvements is necessary before the widespread adoption of such training systems.

## Conclusions

This VR hip arthroscopy simulator was demonstrated to have a sufficient level of aesthetic and tactile verisimilitude, thus establishing its face validity.

## Electronic supplementary material

Below is the link to the electronic supplementary material.

Supplementary material 1. Video 1 Video displaying diagnostic arthroscopy module. Each participant was required to locate a series of twelve consecutive targets within the hip joint using an arthroscope. Six targets were visualized via the anterolateral portal, three via the anterior, and three via the posterolateral. The participants were required to place each target in the centre of the monitor for three seconds before the location of the next target in the examination sequence was displayed to them (MP4 25267 KB)
